# Life Cycle Assessment of Production of Hydrochar via Hydrothermal Carbonization of Date Palm Fronds Biomass

**DOI:** 10.3390/ma16206653

**Published:** 2023-10-11

**Authors:** Chun-Yang Yin, Mohanad El-Harbawi, Zhong-Tao Jiang

**Affiliations:** 1 Newcastle University in Singapore, 537 Clementi Road #06-01, SIT Building @ Ngee Ann Polytechnic, Singapore 599493, Singapore; 2 Chemical Engineering Department, College of Engineering, King Saud University, P.O. Box 800, Riyadh 11421, Saudi Arabia; 3 Surface Analysis and Materials Engineering Research Group, School of Mathematics, Statistics, Chemistry and Physics, College of Science, Technology, Engineering and Mathematics, Murdoch University, 90 South St., Murdoch, WA 6150, Australia; z.jiang@murdoch.edu.au

**Keywords:** life cycle assessment, hydrothermal carbonization, date palm, biomass

## Abstract

This study presents novel life cycle assessment (LCA) findings on hydrochar production from Saudi-Arabia-based date palm fronds biomass waste using hydrothermal carbonization (HTC). The LCA procedure incorporated normalization, weighting, and improvement assessment. The system boundary encompassed water consumption and energy requirements within a lab setting representing a gate-to-gate process. The OpenLCA 1.11.0 software with the European Life Cycle Database 3.2 (ELCD 3.2) was utilized for the study and we employed the ReCiPe Midpoint (H) 2016 and Environmental Footprint 3.0 (EF 3.0) impact assessment methods. The results indicated that fossil fuel usage represented the most significant impact category with the HTC and drying processes identified as major contributors. It was also observed that the HTC process exerted far greater detrimental impacts on the environment than the biomass grinding process. The overwhelming impact of fossil fuel resources could be mitigated by optimizing the batches of biomass or hydrochar samples in each operation, which could alleviate fossil fuel consumption by up to 94%. The findings emphasize the need for targeted interventions to mitigate the environmental burden and contribute to sustainable hydrochar production.

## 1. Introduction

The growing demand for sustainable and renewable energy sources has driven extensive research and development efforts in the field of biomass utilization. Biomass holds great potential as a feedstock for various energy and material applications. In recent years, hydrothermal carbonization (HTC) has emerged as a promising technology for converting biomass into a valuable carbon-rich product known as hydrochar. Date palm (*Phoenix dactylifera*) is a species of flowering plant widely found in tropical and subtropical regions, which offers a significant biomass resource that remains mostly untapped. The utilization of date palm waste biomass through HTC presents an attractive opportunity to not only generate renewable energy but also to address the environmental challenges associated with its disposal and contribute to a more sustainable and circular bioeconomy.

Life Cycle Assessment (LCA) is a widely recognized methodology used to evaluate the environmental impacts of products and processes throughout their entire life cycle. By quantifying and assessing various environmental indicators, LCA provides valuable insights into the environmental performance of different technologies and can aid in decision-making towards more sustainable practices. Indeed, LCA is a vital decision-support method that enables companies to benchmark and optimize the environmental performance of products as well as for relevant stakeholders (e.g., governments) to design policies for sustainable consumption and creation [1]. In terms of agricultural-based industries, van der Werf and co-researchers [2] indicated that current LCA techniques and findings are inclined to favour high-input intensive agricultural systems and misrepresent less-intensive agroecological systems such as organic agriculture. It is our opinion that date palm plantation can be categorized as a relatively less-intensive agroecological system and, as such, our current LCA analysis would add value to the overall LCA literature. 

Benavente et al. [3] employed LCA to evaluate the environmental impacts associated with the use of HTC to treat olive mill waste and compared the results with the life cycle assessment of aerobic composting, anaerobic digestion and incineration. Zhang and co-researchers [4] used the life cycle assessment (LCA) approach to evaluate the environmental impact of generating 1 MJ of electricity from sugarcane bagasse hydrochar. Berge and co-workers [5] evaluated the environmental impact of energy production from hydrochar obtained through the hydrothermal carbonization of food waste. Corvalán and co-researchers [6] utilized the LCA and compared the environmental impacts between the HTC and gasification processes. Readers are referred to the review paper published by Mayer et al. [7] for further details on the LCA of waste-to-energy. Shaheen and co-researchers [8] conducted a LCA on UAE-based date palm waste biochar synthesized using pyrolysis and compared its environmental impacts and adsorption effectiveness with activated carbon produced from woody debris. Similarly, Thornley and co-researchers [9] utilized LCA and indicated that biochar production systems can potentially deliver the highest greenhouse gas reductions per unit area of land in comparison with other bioenergy systems (electricity, heat and chemical). Recently, Gallego-Ramírez and co-researchers [10] conducted a life cycle assessment (LCA) on the generation of Pinus patula raw biochar and discovered that the predominant impacts were attributed to the generation of gases and polycyclic aromatic hydrocarbons.

In our previous studies [11,12], we investigated the potential of palm-based biomass as a feedstock for the production of a type of hydrochar, i.e., carbon microspheres through hydrothermal carbonization (HTC). It should be noted that hydrochar is synthesized from a slurry (i.e., a two-phase mixture of solid and liquid) using HTC while biochar can be synthesized as a solid by-product material in a dry carbonization process such as pyrolysis [13]. One of our previous studies [10] focused on utilizing the carbon microspheres for the adsorption of methylene blue, a common dye pollutant found in wastewater. The results highlighted the effectiveness of the carbon microspheres derived from palm biomass in removing methylene blue from aqueous solutions. This work demonstrated the potential of palm biomass as a sustainable precursor for producing carbon-based adsorbents. 

Building upon the findings of our previous research, the current study aims to evaluate the environmental impacts associated with the HTC process applied to date palm fronds biomass. The fronds are essentially the leaf or leaf-like component of a palm. In Saudi Arabia, palm date wastes (fronds, offshoots, dried fronds base and date pits) are generated from date palm cultivation with approximately 20 kg of waste produced per year from a single date palm [14] (Faiad et al., 2022). While a portion is burned or left to decompose naturally, a small fraction is used as animal feed or fertilizer. By employing a LCA approach, the research will provide a comprehensive analysis of the environmental performance of the date palm HTC process. To the best of our knowledge, there is no prior LCA study conducted on a date palm HTC process. We consider a wide range of impact categories, including greenhouse gas emissions, energy consumption and water usage, with the aim of providing a holistic understanding of the environmental profile of the date palm waste HTC process.

## 2. Methodology

### 2.1. Description of Waste Palm HTC Process

Date palm fronds were collected from a local palm farm in Riyadh, Saudi Arabia. An amount of 1 kg of date palm fronds was washed with approximately 3 L of water to remove dirt and dust. The palm waste biomass was subsequently dried in a laboratory dryer at 80 °C for 48 h. A Fritsch Pulverisette 15 cutting mill was used to grind the biomass into powder. NL Scientific sieve shaker, model No. NL1015X/001, was operated for 20 min to obtain the desired grind size (0.25 mm). 

A total of 2.5 g of the ground palm waste was added to 25 mL of deionized water in a 50 mL flask and stirred magnetically for 2 h. The mixed material was then placed in a 45 mL Teflon tube PARR digestion vessel and sealed tightly. The vessel was then heated in a muffle furnace at 230 °C for 8 h. After completion of the HTC process, the PARR vessel was withdrawn from the muffle furnace and cooled at room temperature for approximately 6 h. The dark brown liquid resulting from the HTC process was washed with 2 L of deionized water via a vacuum filtration pump. A Büchner funnel glass filter with additional filter paper (Macherey Nagel filter paper MN 616) was used to ensure that no solid products were lost during filtration. The filter paper, which contained the wet solids cake, was removed from the Büchner funnel and kept in a drying oven at 80 °C for 24 h. The solid material adhering to the filter paper was scraped off and then ground manually into a soft powder using a ceramic/glass mortar. Finally, the hydrochar is stored in a well-sealed container in a dry place. The Tuscan VEGA II LSU (Tuscan Inc., Tucson, AZ, USA) scanning electron microscopy (SEM) was used to study the surface morphology of the carbon microspheres present in the hydrochar. 

Hydrolysis, dehydration, decarboxylation, condensation polymerization and aromatization are the major reactions that occur during HTC processes [15]. Removing carboxyl and -OH groups substantially reduces the O/C and the H/C atomic ratios to render the final product HTC “denser” [16] (Romano et al., 2023). In our HTC process [10], we observed that it followed a trend corresponding to a dehydration process with a minor occurrence of the decarboxylation process. 

### 2.2. Life Cycle Assessment Methodology

The ISO 14040 standard was used as framework for conducting the LCA for the current study comprising four main stages, namely, goal and scope definition, life cycle inventory analysis (LCI), life cycle impact assessment (LCIA) and interpretation.

#### 2.2.1. Goal and Scope Definition

**Figure 1:**
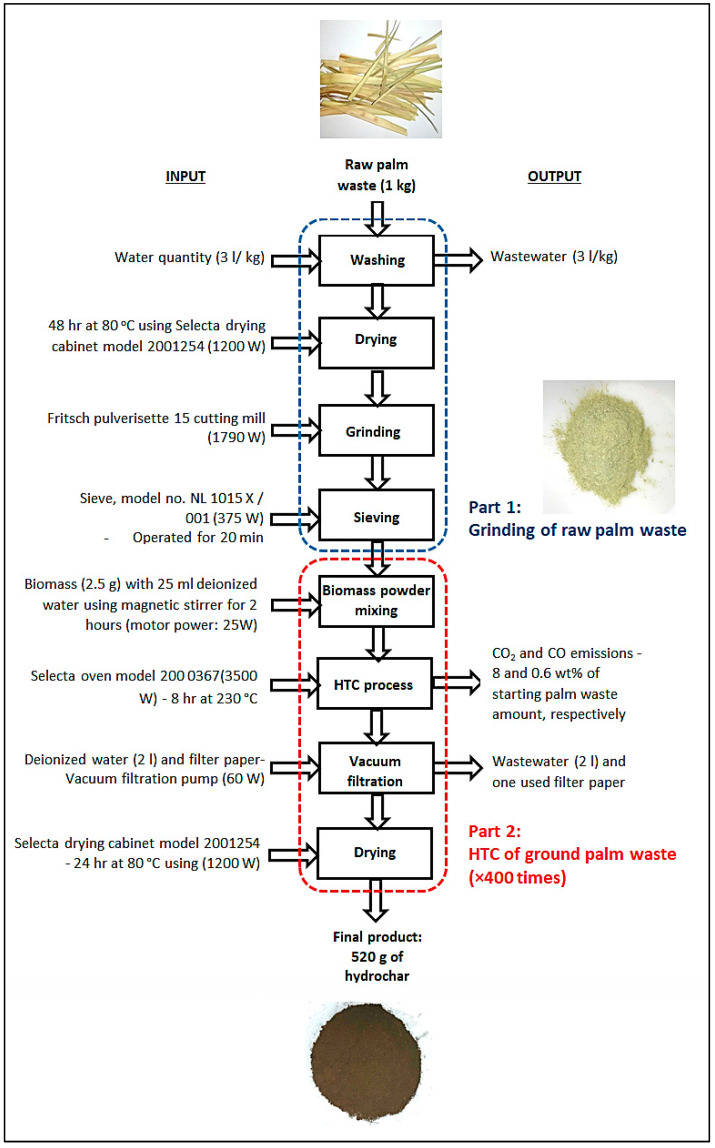
Process flow diagram of the hydrochar production process from raw date palm biomass with corresponding input and output components.

The use (i.e., processing) of 1 kg of palm waste biomass was selected as the functional unit for the current LCA study. The system boundary encompassed the water consumption and energy required to produce the hydrochar within a lab setting while the palm waste transportation process was excluded (i.e., gate-to-gate process). As such, the LCA scope includes the analysis of the reception of the raw palm waste to the conclusion of the HTC process. Two main sections of unit operations were delineated in the study, i.e., the grinding of raw palm waste and HTC of ground palm waste for comparison purposes. Figure 1 shows the process flow diagram of the hydrochar production process from raw date palm biomass with corresponding input and output components.

There are several assumptions used in conducting the present LCA study. The amount of water evaporated from the drying process was negligible compared to the water required for the washing process. In terms of accounting for the consumption of energy in the process, we used a % mix of natural gas and oil of 61% and 39% to represent energy use—this % mix was obtained from a 2021 dataset reported by Ember (https://ember-climate.org/countries-and-regions/countries/saudi-arabia/ accessed on 15 July 2023). We used 8 and 0.6 wt% of starting palm waste amounts to represent the amount of CO_2_ and CO released as a result of the HTC process—these assumed values were based on a prior biomass HTC study by Hoekman and co-researchers [17] and duly adopted in the current study. To ensure consistency in the functional unit, the input and output values for the second half of the process (i.e., HTC process) were multiplied 400 times to equate to the functional unit, i.e., 1000 g (1 kg) of biomass used. We assumed that the embodied energy of the utilized technologies is negligible based on the reported study by Výtisk and co-researchers [18] because such systems possessed very low embodied energy compared to HTC operational energy use. The origin of water resources was stipulated to be in the ground by Gulf Cooperation Council because it is crucial to note that Saudi Arabia, like many other arid and desert regions, heavily relies on groundwater for its water supply due to limited surface water resources.

#### 2.2.2. Life Cycle Inventory Analysis (LCI)

This stage involves the compilation of a comprehensive inventory of all inputs (energy, utilities, etc.) and outputs (emissions, waste, etc.) with respect to our biomass-based process confined within specific boundaries. This encompasses a collection of data on all pertinent processes including washing, drying, grinding, sieving, mixing, HTC, filtration and final drying. Inventory data concerning energy and water consumption were established based on lab-scale experiments which were conducted in our prior study [11]. The openLCA software (version 1.11.0) was used whereby the European Life Cycle Database 3.2 (ELCD 3.2) was incorporated into the software and used for our current study. The “geographical location” in the software was indicated as “Saudi Arabia”. 

### 2.3. Life Cycle Impact Assessment (LCIA) 

In this stage, the environmental impacts associated with the inputs and outputs identified in the LCI stage were assessed. Two impact assessment methods were used, namely, the ReCiPe Midpoint (H) 2016 and Environmental Footprint 3.0 (EF 3.0) [19] methods. The ReCiPe Midpoint (H) 2016 method, which has 18 impact categories, was used to change water and energy consumption into environmental impacts [20,21]. This is a widely researched and utilized LCIA method. The Midpoint method for ReCiPe is selected over the Endpoint method because the former affords more depth of information (rather than breath) with lower statistical uncertainties. EF 3.0 is a method that aims at assessing the environmental impacts of products and organisations through midpoint impact categories including the toxicity-related impacts [22] established by the European Commission (CE) via the Product Environmental Footprint initiative. 

The EF 3.0 method considers a wide-ranging list of impact categories including acidification, climate change (biogenic, fossil or land use), ecotoxicity, freshwater (inorganics, metals or organics), eutrophication (freshwater, marine or terrestrial), human toxicity (cancer or non-cancer), ionizing radiation, land use, ozone depletion, particulate matter, photochemical ozone formation, resource use (fossils or minerals/metals) and water use. We also considered another method, the Eco-Indicator 99 [23] but decided to select EF 3.0 to accompany the use of ReCiPe instead because EF 3.0 is more up-to-date and considers more impact categories with various sub-categories. The selection of both the ReCiPe and EF 3.0 methods in the study is intended to be complementary, given that the former predominantly focuses on the assessment of potential impacts in a limited number of environmental categories, though with a detailed analysis of specific environmental factors. In contrast, EF 3.0 encompasses a broader perspective, covering a wider range of environmental impact categories to afford a more all-inclusive understanding of the overall environmental footprint.

### 2.4. Life Cycle Interpretation 

The results of the previous stages were analyzed, in which the environmental implications of the assessed processes were discussed prior to the identification of potential improvement opportunities. The interpretation stage in LCA is a vital step that incorporates analyzing and drawing conclusions from the LCA results. In the current study, the LCA results pertaining to the two main sections of unit operations (grinding and HTC) are comprehensively examined and analyzed to identify significant environmental impacts and hotspots within their life cycle boundaries.

### 2.5. Normalization and Weighting 

We conducted normalization and weighting for our LCA datasets. Normalization is the process of comparing the results of different impact categories in an LCA study. It provides a relative perspective on the environmental performance of varying systems by utilizing a common reference point. This reference point is normally derived from the average environmental impact of a specific region, industry or even a global baseline. Weighting is the process of assigning relative importance or significance to different impact categories based on stakeholders’ values or preferences. The ReCiPe Midpoint (H) 2016 method has an in-built World (2010) H global database, while for the EF 3.0 LCIA method, the EF 3.0 normalization and weighting set was integrated in our study for normalization and weighting purposes.

## 3. Results and Discussion

### 3.1. Hydrochar and Presence of Carbon Microspheres

**Figure 2:**
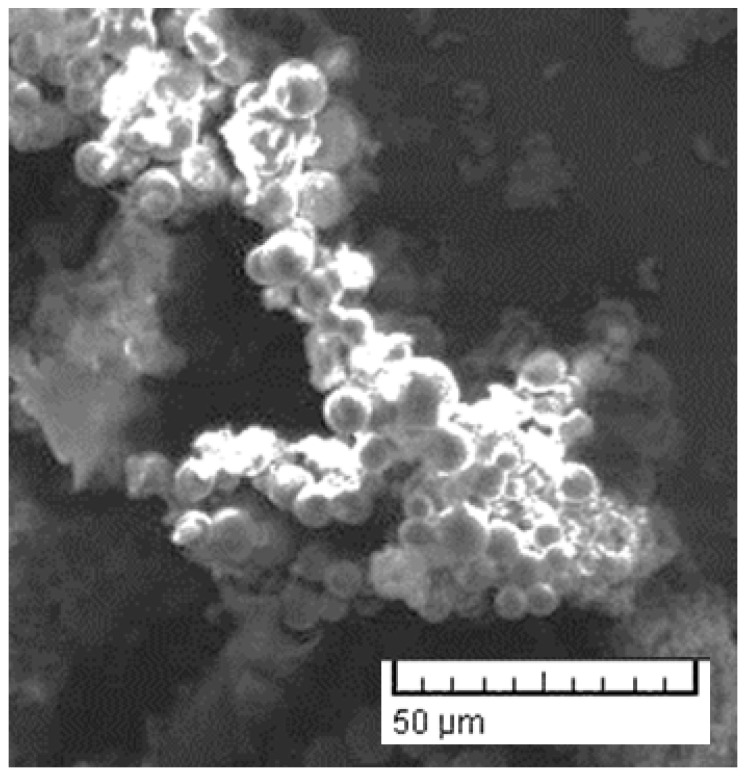
Scanning electron micrograph of carbon microspheres in the hydrochar.

Figure 2 shows the scanning electron micrograph of carbon microspheres in the hydrochar. These micrographs are used to ascertain the surface properties of the microspheres which, in turn, affect the emissions of volatile gases, including CO_2_, from the hydrochar. There are noticeable microspheres with approximate sizes ranging from 3 to 10 microns with no discernible porosity. The morphology, texture and the shape of the microspheres are similar to our previous work [11,12] and those synthesized using monosaccharides by other researchers [24,25]. During the HTC process, carbon from the date palm biomass is retained in the synthesized carbonaceous material. This facilitates the sequestration of carbon, thereby inhibiting its release into the atmosphere as a greenhouse gas [26]. By locking carbon in a stable solid form, HTC contributes to mitigating climate change by reducing CO_2_ emissions, even though it should be noted that trace CO_2_ is emitted from the HTC decarboxylation process.

### 3.2. LCA Results

Table 1 shows the impact scores for the current process involving two main sections of unit operations, i.e., the grinding of raw palm waste and HTC of ground palm waste as observed in the LCIA stage. Essentially, impact scores in the LCA are utilized to quantify the magnitude of environmental impacts within each impact category. It should be noted that the impact scores assigned to each category generally represent the magnitude of the impacts in relation to a reference or baseline scenario. These scores can be expressed in various units, as detailed in Table 1. Three impact categories (*fossil resource scarcity, global warming* and *water consumption*) are assigned scores by ReCiPe, while five impact categories (*resource use, fossils, climate change, water use, ecotoxicity, freshwater* and *human toxicity, non-cancer*) by EF 3.0. Out of the many impact categories considered using both methods, only the aforestated impact categories were selected for the ReCiPe and EF 3.0, respectively, as they possessed values/scores (i.e., non-zero) as detected in the OpenLCA 1.11.0 software, indicating that they exhibited at least some form of impact.

It can be seen that all the impact scores for Part 2 of the process (i.e., HTC) are substantially greater than the scores for Part 1 of the process (i.e., grinding) for all corresponding categories. This indicates that the HTC process exerts far greater detrimental impacts on the environment than the grinding process. For example, the energy use associated with Saudi-based fossil fuel use for Part 2 (HTC) is observed to be in excess of 300 times higher than that of Part 1 (grinding), while water use/consumption for Part 2 is in excess of 200 times higher than Part 1. The marginal impact scores associated with the Part 1 process are somewhat expected since it only involves mostly preparatory processes such as washing and biomass size reduction via grinding. The marginal impact scores observed for *global warming* and *climate change* for the two methods are attributed to the direct emission of CO_2_ and CO, possibly from the decarboxylation process that occurs during the HTC process [15,17]. According to the EF 3.0 method/database in terms of flow impact contributions, carbon monoxide exerts an impact on climate change with a score of 0.00942 kg CO_2_ eq. (10.54%) in comparison to 0.08 kg CO_2_ eq. (89.46%) from carbon dioxide for the HTC process. This is because, according to IPCC [27], carbon monoxide is an *indirect* greenhouse gas with a radiative forcing nearly twice that of carbon dioxide on a molecular basis. Similarly, the minimal impact scores observed for *ecotoxicity*, *freshwater* and *human toxicity*, *non-cancer* are attributed to the direct emission of CO from the HTC process. At this point, it would be difficult to compare the relative impact of a category with another category as the units are not directly equivalent in terms of magnitude and definition. 

To facilitate a fairer comparison between the impact categories, we have conducted the normalization stage. Normalization theoretically addresses differences in category units of measurement or magnitudes by normalizing the results via a common reference value, preventing the biases that may arise from comparing impacts that vary widely in scale. The reference values should represent the average or best available data for a specific geographical region. For example, in our current study, ReCiPe uses world data for normalization. 

**Figure 3:**
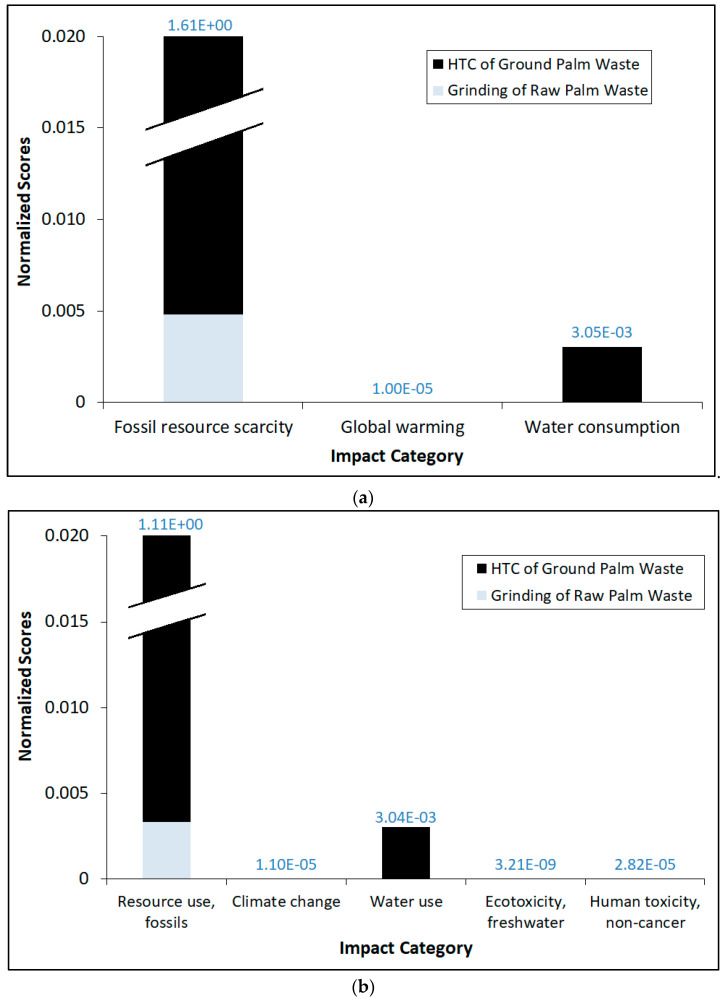
Comparative normalized impact category scores by using (**a**) ReCiPe 2016 Midpoint (H); and (**b**) EF 3.0 impact assessment methods for the entire process.

**Figure 4:**
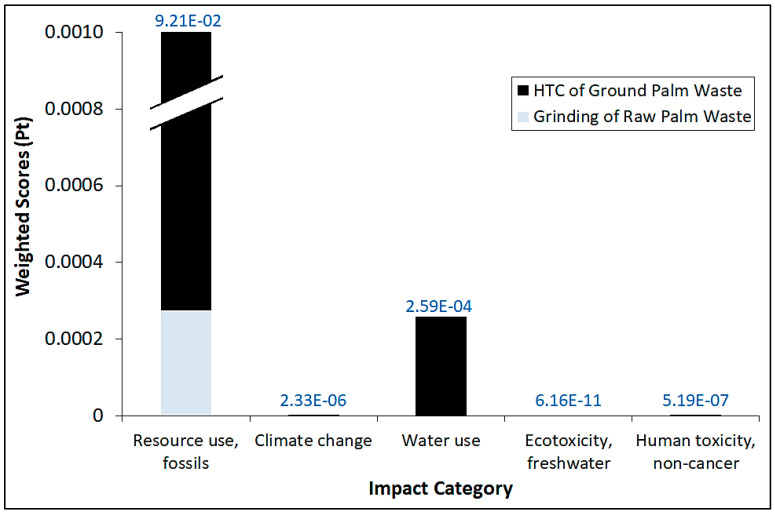
Comparative weighted impact category scores by using EF 3.0 impact assessment methods for the entire process.

Figure 3 shows the comparative normalized impact category scores by using both ReCiPe 2016 Midpoint (H) and EF 3.0 impact assessment methods for the entire process. It should be noted that the data values on top of the bar refer to the total normalized scores for each category. Interestingly, the overall trend of normalized scores generally mirrors their corresponding impact scores. Indeed, the use of fossil fuel resources overwhelmingly dominates over other impact categories, even after normalization for both methods. Water use/consumption normalized scores are a distant second compared to fossil fuel use, while very nominal normalized scores are observed for the other impact categories. Again, even after normalization, the impacts exerted by the Part 1 (grinding) process are significantly lower than those for Part 2 in terms of magnitude. We also conducted weighting of the impact scores by using the EF 3.0 method and the results are shown in Figure 4, in which the data values on top of the bar refer to the total weighted scores for each category. Similarly, the overall trend of the weighted scores generally mirrors their corresponding normalized scores, whereby the utilization of fossil fuel resources outweighs climate change, ecotoxicity and human toxicity in the case of EF 3.0. The impact attributed to ecotoxicity can be construed to be very marginal in comparison to even the nominal weighted values of climate change and human toxicity. It is essential to note that both normalization and weighting can involve value judgments and stakeholder preferences. As such, the transparency and inclusiveness of the decision-making process in establishing biomass-based reference values and weighting factors are important to ensure the credibility and acceptance of LCA results.

### 3.3. Improvement Assessment

We subsequently identified the evaluated potential improvement options based on the obtained results and interpretation. The aim of this LCA stage is to explore strategies for reducing the negative impacts and enhancing the overall sustainability performance of the grinding/HTC processes. The LCA results discussed thus far involve the assumption of the use of a single PARR digestion vessel with a subsequent single use of the oven and dryer multiplied by 400 times to equate to the functional unit, i.e., 1000 g (1 kg) of biomass used. 

**Figure 5:**
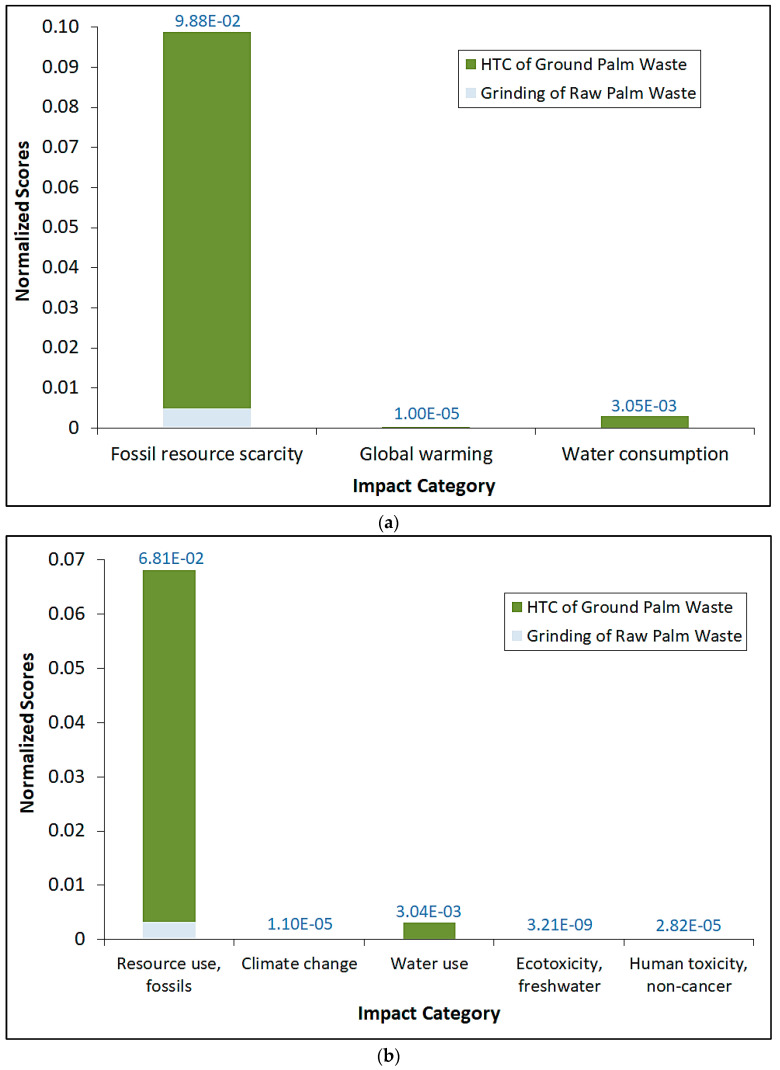
Comparative normalized impact category scores by using (**a**) ReCiPe 2016 Midpoint (H); and (**b**) EF 3.0 impact assessment methods for the entire process with reduced oven and drying cabinet usage.

**Figure 6:**
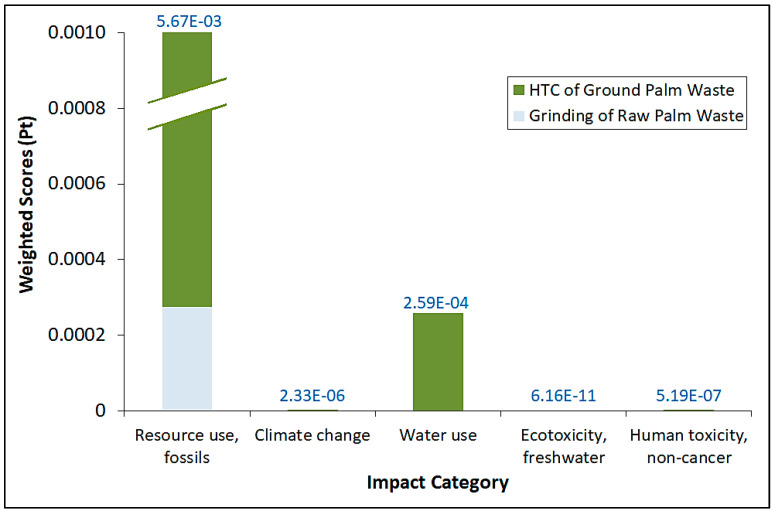
Comparative weighted impact category scores by using EF 3.0 impact assessment methods for the entire process with reduced oven and drying cabinet usage.

Our results indicate the overwhelming impact of fossil fuel resource use attributed to the two main pieces of equipment, namely, the oven and drying cabinet—this could be mitigated by optimizing the batches of biomass or hydrochar samples in each operation. As such, we could intuitively reduce the energy requirement by concurrently using multiple PARR digestion vessels in oven operation and conducting multiple samples drying in the drying cabinet. Based on the size of the oven and drying cabinet, we estimated that we could reduce the use of the oven from 400 to 45 times (nine PARR vessels could fit into the oven) while the use of the drying cabinet could be reduced from 400 to 7 times (about 60 post-filtration hydrochar batches could fit into the cabinet). By incorporating these reductions, we discover that the HTC process impact scores for *fossil resource scarcity* and *resource use, fossils* have been reduced from 1569.58 kg oil eq. and 71,798.40 MJ to 92.12 kg oil eq. and 4214.16 MJ, respectively. These constitute decreases of about 94% of energy use attributed to fossil fuel. Similarly, these reductions can also be observed for the new normalized (Figure 5) and weighted (Figure 6) impact scores which incorporate reduced oven and drying cabinet usage. It should be noted that the data values on top of the bar refer to the total normalized and weighted scores for each category, respectively.

## 4. Conclusions

In this study, we conducted a comprehensive LCA study to assess the environmental impacts associated with the production of hydrochar from Saudi-Arabia-based date palm fronds biomass waste using HTC. The LCA procedure included normalization, weighting and improvement assessment, thereby providing important insights into the sustainability of this process. Our findings revealed that *fossil resource scarcity*/*resource use, fossils* emerged as the most significant impact category throughout the HTC process. This result highlights the need for targeted interventions to mitigate the environmental burden associated with this aspect of the production cycle. Through our analysis, we identified the oven and drying cabinet as the primary sources of environmental burden in the HTC process, thus allowing for targeted optimization efforts to minimize energy requirements. The implementation of energy-efficient alternatives or renewable energy sources for these specific stages could significantly enhance the overall sustainability of the hydrochar production process. By quantifying the environmental impacts associated with the HTC of date palm fronds biomass waste, our study affords valuable information for policymakers, industry stakeholders and researchers seeking to promote sustainable waste management and resource recovery strategies in the Middle East region. The results underscore the importance of adopting a life cycle perspective when evaluating the environmental implications of any production process, enabling a more holistic and informed decision-making process. Future research should focus on investigating the potential synergies and trade-offs between hydrochar production and other environmental impact categories to facilitate a more comprehensive understanding of the overall sustainability of this process.

## Figures and Tables

**Table 1:** Impact scores for current process—grinding of raw palm waste and HTC of ground palm waste.

	Impact Category	Impact Assessment Method	Score
Grinding of raw palm waste	Fossil resource scarcity	ReCiPe Midpoint (H) 2016	4.71 kg oil eq.
	Global warming	ReCiPe Midpoint (H) 2016	Not observed in situ
	Water consumption	ReCiPe Midpoint (H) 2016	0.003 m^3^
	Resource use, fossils	Environmental Footprint 3.0	215.33 MJ
	Climate change	Environmental Footprint 3.0	Not observed in situ
	Water use	Environmental Footprint 3.0	0.129 m^3^ depriv.
	Ecotoxicity, freshwater	Environmental Footprint 3.0	Not observed in situ
	Human toxicity, non-cancer	Environmental Footprint 3.0	Not observed in situ
HTC of ground palm waste	Fossil resource scarcity	ReCiPe Midpoint (H) 2016	1569.58 kg oil eq.
	Global warming	ReCiPe Midpoint (H) 2016	0.08 kg CO_2_ eq.
	Water consumption	ReCiPe Midpoint (H) 2016	0.81 m^3^
	Resource use, fossils	Environmental Footprint 3.0	71,798.40 MJ
	Climate change	Environmental Footprint 3.0	0.0894 kg CO_2_ eq. ^#^
	Water use	Environmental Footprint 3.0	34.79 m^3^ depriv.
	Ecotoxicity, freshwater	Environmental Footprint 3.0	0.000137 CTU_e_ *
	Human toxicity, non-cancer	Environmental Footprint 3.0	6.48 × 10^−9^ CTU_h_ *

* CTU_e_ and CTU_h_ refer to comparative toxic unit for ecosystem and comparative toxic unit for humans, respectively. ^#^ Score consists of 0.00942 kg CO_2_ eq. (10.54%) from CO and 0.08 kg CO_2_ eq. (89.46%) from CO_2_ for the HTC process.

## Data Availability

Not applicable.
